# Evaluation of Selected Culinary-Medicinal Mushrooms for Antioxidant and ACE Inhibitory Activities

**DOI:** 10.1155/2012/464238

**Published:** 2011-06-18

**Authors:** Noorlidah Abdullah, Siti Marjiana Ismail, Norhaniza Aminudin, Adawiyah Suriza Shuib, Beng Fye Lau

**Affiliations:** Mushroom Research Centre, Institute of Biological Sciences, Faculty of Science, University of Malaya, 50603 Kuala Lumpur, Malaysia

## Abstract

Considering the importance of diet in prevention of oxidative stress-related diseases including hypertension, this study was undertaken to evaluate the *in vitro* antioxidant and ACE inhibitory activities of selected culinary-medicinal mushrooms extracted by boiling in water for 30 min. Antioxidant capacity was measured using the following assays: DPPH free radical scavenging activity, *β*-carotene bleaching, inhibition of lipid peroxidation, reducing power ability, and cupric ion reducing antioxidant capacity (CUPRAC). Antioxidant potential of each mushroom species was calculated based on the average percentages relative to quercetin and summarized as Antioxidant Index (AI). *Ganoderma lucidum* (30.1%), *Schizophyllum commune* (27.6%), and *Hericium erinaceus* (17.7%) showed relatively high AI. Total phenolics in these mushrooms varied between 6.19 to 63.51 mg GAE/g extract. In the ACE inhibitory assay, *G. lucidum* was shown to be the most potent species (IC_50_ = 50 *μ*g/mL). Based on our findings, culinary-medicinal mushrooms can be considered as potential source of dietary antioxidant and ACE inhibitory agents.

## 1. Introduction

Reactive oxygen species (ROS) have been implicated in food deterioration as well as pathogenesis of various human diseases and aging-related disorders [1]. Epidemiological studies have revealed the close association between intake of food rich in antioxidants such as vegetables, fruits, and cereals with prevention of the aforementioned pathologies [2]. Intake of exogenous antioxidants is crucial to maintain an adequate level of antioxidants in order to balance the ROS especially when human *in vivo *antioxidant defence and repair systems are considered to be insufficient to totally prevent the damage. Oxidative stress has been linked to hypertension—imbalance in superoxide and nitric oxide production will lead to reduced vasodilation [3]. If hypertension persists for a long period, it could be one of the risk factors for strokes, heart diseases, and eventually leading to chronic renal failure. 

Synthetic antioxidants that are widely used in the food industry such as butylated hydroxyanisole (BHA), butylated hydroxytoluene (BHT), and *tert-*butylated hydroxyquinine (TBHQ) have been reported to be carcinogenic; thus, their use has been restricted [4]. Treatments by administration of antihypertensive drugs, for example, inhibitors of the angiotensin I-converting enzyme (ACE) aim to reduce blood pressure and lower the risk of hypertension complications. However, the use of synthetic ACE inhibitors such as captopril and enalapril caused serious side effects including dry cough, skin rashes, and allergic reactions [5]. This situation has prompted the search for potential antioxidant and ACE inhibitors from natural sources. 

Combination of both biological activities in one multifunctional preparation, especially in food, was suggested to be useful for controlling the risk of cardiovascular diseases [6]. In fact, Münzel and Keaney [3] proposed that ACE inhibitors possess novel antioxidant strategy by improving vasoconstriction, increasing bioactivity of nitric oxide, and inhibiting vascular superoxide production at its source. Therefore, consumption of antioxidant-rich foods which possess ACE inhibitory activity can be considered as an alternative therapy for treatment of hypertension especially for prehypertensive patients whose blood pressure is marginally or mildly high but not high enough to warrant the prescription of blood-pressure-lowering medications as suggested by Chen et al. [7].

Mushrooms are considered as functional food with physiological beneficial constituents [1] and consumption of several mushrooms such as *Agaricus bisporus*, *Lentinula edodes,* and *Pleurotus* spp. has become popular over the years. The broad medicinal values of mushrooms have a promising future as a branch of alternative medicine [8] and hence, scientific research directed to validation of claimed medicinal properties is vital. Mushroom decoctions in folk medicine often involve the use of hot water to extract soluble components from the fruiting body. Accordingly, crushed or small pieces of the fruiting body are boiled and the resulting decoction is consumed. Besides that, mushrooms are usually not eaten raw but subjected to various food processing procedures so that they will be more readily assimulated by digestion [9]. Hence, it can be said that preparation of hot water extracts similate cooking conditions—the typical way of how edible mushrooms are consumed.

Information obtained from analysis using hot water extracts was considered a better indicator of biological activities especially upon consumption [10, 11]. Since most reports on biological properties of mushrooms utilised various organic solvents for preparation of extracts, it is rather difficult to make comparisons between different laboratories taking into account variation in the procedures. Evaluation of several mushroom species using the same set of procedures is vital for accurate and fair comparison of biological activities studied. Hence, the objective of this study is to analyse the *in vitro* antioxidant and ACE inhibitory activities of hot water extracts of selected culinary-medicinal mushrooms.

## 2. Materials and Methods

### 2.1. Mushroom Collection

Fourteen species of culinary-medicinal mushrooms used in this study (Table 1) were obtained from mushroom farms and supermarkets in Selangor, Malaysia. The samples were identified and authenticated by experts in the Mushroom Research Centre, University of Malaya; voucher specimens were deposited in the University of Malaya herbarium (KLU).

### 2.2. Preparation of Mushroom Hot Water Extracts

All mushroom fruiting bodies were cleaned, cut into smaller pieces, and boiled in distilled water at the ratio of 1 : 10 (w/v) at 100°C for 30 min. Boiled mushrooms were cooled to room temperature, removed by using Whatman No. 1 filter paper and hot water extracts obtained were freeze-dried (Labconco). The extracts were kept in desiccator at room temperature for further analysis.

### 2.3. Estimation of Total Phenolic Content

Total phenolic content of the mushroom extracts was estimated using Folin-Ciocalteu reagent according to the method of Slinkard and Singleton [12] with some modifications. Initially, 250 *μ*L of each mushroom extract was mixed with 250 *μ*L of 10% Folin-Ciocalteu reagent, followed with the addition of 500 *μ*L of saturated sodium carbonate (10% aqueous solution) after 2 min of incubation at room temperature. The mixture was kept in the dark for 1 h before absorbance was taken at 750 nm. A calibration curve using gallic acid (2–10 *μ*g/mL) was prepared. Total phenolic content of the mushroom extracts was expressed as gallic acid equivalents (GAEs), which reflect the phenolic content as the amount of gallic acid (mg) in 1 g of extract.

### 2.4. Antioxidant Capacity Assays

In all assays, quercetin, butylated hydroxyanisole (BHA), and ascorbic acid were used as positive control.

### 2.5. Scavenging Effect on 1,1-Diphenyl-2-Picrylhydrazyl (DPPH) Radicals

The DPPH free radical scavenging activity of the extracts was measured according to the method of Brand-Williams et al. [13]. Stock solution of each mushroom extract (50 mg/mL) was diluted to a concentration in the range of 0.1 to 50 mg/mL. For the test, 3.9 mL of 0.06 mM DPPH radical (Sigma) was added to 0.1 mL of mushroom extract. Reaction mixture was vortexed and absorbance was measured at 515 nm using a spectrophotometer with methanol as the blank. The decrease in absorbance was monitored at 0 min, 1 min, 2 min, and every 15 min until the reaction has reached a plateau. The time taken to reach the steady state was determined by one-way analysis of variance (ANOVA). The DPPH free radical scavenging activity, expressed as percentage of radical scavenging activity, was calculated as follows:

(1) $$\;\text{Radical}\;\text{scavenging}\;\text{activity}\;\left(\text{SA}\right)=\frac{A_{0}-A_{s}}{A_{0}}\times 100,$$

where *A_0_* is the absorbance of 0.06 mM methanolic DPPH only whereas *A_s_* is the absorbance of the reaction mixture.

### 2.6. *β*-Carotene Bleaching Activity

*β*-carotene bleaching activity of the mushroom extracts was evaluated using *β*-carotene-linoleic acid model system previously described [14] with some modifications. Briefly, 2 mg of *β*-carotene in 10 mL chloroform was mixed with 40 *μ*L of linoleic acid and 400 *μ*L of Tween 80 emulsifier. Chloroform was allowed to evaporate under vacuum, prior to addition of 100 mL of distilled water with vigorous shaking. Then, 4.8 mL of the mixture was transferred into tubes containing different concentrations of extract. Upon addition of the emulsion into each tube, the zero time absorbance was measured at 470 nm using a spectrophotometer. The emulsion system was incubated for 2 h at 50°C.

### 2.7. Inhibition of Lipid Peroxidation of Buffered Egg Yolk

The ability of mushroom extracts to inhibit lipid peroxidation was determined according to the method of Daker et al. [15]. The reaction mixture contained 1 mL of fowl egg yolk emulsified with 0.1 M phosphate buffer (pH 7.4) to obtain a final concentration of 25 g/L and 100 *μ*L of 1000 *μ*M Fe^2+^. The stock solution of each mushroom extract (360 mg/mL) was prepared and then diluted to final extract concentrations of 0.1–30 mg/mL. The mixture was incubated at 37°C for 1 h before being treated with 0.5 mL of freshly prepared 15% trichloroacetic acid (TCA) and 1.0 mL of 1% thiobarbituric acid (TBA). The reaction tubes were further incubated in boiling water bath for 10 min. Once cooled to room temperature, the tubes were centrifuged at 3500 × g for 10 min to remove precipitated protein. Finally, 100 *μ*L of supernatant was subjected to an absorbance reading at 532 nm to measure the formation of thiobarbituric acid reactive substances (TBARS). Buffered egg with Fe^2+^ only was used as control in this assay. The percentage inhibition was calculated from the following equation:

(2) $$\text{Inhibition (\%)}=\frac{A_{0}-A_{s}}{A_{0}}\times 100,$$

where *A_0_* is the absorbance of the control whereas *A_s_* is the absorbance of the sample.

### 2.8. Reducing Power Ability

Reducing power of the mushroom extracts was determined according to the method of Öztürk et al. [16]. Diluted mushroom extracts were mixed with 2.5 mL of 0.2 M phosphate buffer (pH 6.6) and 2.5 mL of 1% potassium ferricyanide. Reaction mixtures were incubated at 50°C for 20 min. After the addition of 2.5 mL of 10% TCA, the mixture were centrifuged for 10 min at 1 000 rpm. Then, 2.5 mL of the supernatant were mixed with 2.5 mL of distilled water and 0.5 mL of 0.1% ferric chloride. Absorbance was measured at 700 nm against a blank.

### 2.9. Cupric-Ion-Reducing Antioxidant Capacity (CUPRAC)

CUPRAC assay was performed according to the method by Öztürk et al. [16] with some modifications. The test mixture contained 1 mL of 10 mM of copper (II), 7.5 mM neocuproine, and 1 M ammonium acetate buffer (pH 7.0). Briefly, 1 mL of diluted mushroom extracts in the concentration range of 0.1–20 mg/mL were added to the reaction tubes to achieve final volume of 4 mL. The tubes were incubated for 30 min at room temperature before absorbance at 450 nm was recorded against a blank.

### 2.10. Antioxidant Index (AI)

The Antioxidant Index as proposed by Puttaraju et al. [17] was used to grade the selected culinary-medicinal mushrooms on the basis of their antioxidant potential. Mushroom extracts were graded in a numerical scale based on quercetin which is considered to be equivalent to 100 (Table 5). AI represents the average relative percentages compared to quercetin obtained using five different methodologies for evaluation of antioxidant capacity described above.

### 2.11. ACE Inhibitory Assay

The ACE inhibitory activity assay was performed according to the method by Nakamura et al. [18] with some modifications. Briefly, 200 *μ*L of 5 mM hippuryl-L-histidine-L-leucine (HHL) (Sigma) solution was mixed with 50 *μ*L of each mushroom extract and the mixture was preincubated at 37°C for 3 min. The reaction was initiated by the addition of 20 *μ*L of 0.1 U/mL ACE (Sigma) solution and the mixture was again incubated at 37°C for 30 min. The reaction was terminated with the addition of 250 *μ*L of 1 N HCl. Then, 1.5 mL of ethyl acetate was added to extract the hippuric acid liberated by the reaction. The solution was centrifuged for 10 min; the ethyl acetate layer was then aspirated and evaporated under vacuum condition. The dried hippuric acid was redissolved in 1 mL of distilled water and measured spectrophotometrically at 228 nm. The ACE inhibitory activity of the mushroom extracts was determined by the following equation:

(3) $$\text{Percentage}\;\text{of}\;\text{inhibition}\;\left(\%\right)=\frac{\text{B}-\text{A}}{\text{B}-\text{C}}\times 100,$$

where *A* is the absorbance of ACE and mushroom extracts, *B* is the absorbance of ACE and HHL and *C* is the absorbance of HHL only.

### 2.12. Statistical Analysis

All analysis was performed in triplicates and data was recorded as means ± standard deviation. Analysis was carried out using one-way analysis of variance (ANOVA) in Statgraphics Plus for Windows 3.0. The test stated any significant differences between the means at 95% (*P* < .05) level were considered as statistically significant. Correlation and regression analysis were carried out using the Microsoft EXCEL.

## 3. Results and Discussion

### 3.1. Estimation of Total Phenolic Content

Considering the physiological importance of phenolic compounds and their contribution towards total antioxidant capacity, Folin-Ciocalteu method was used to estimate the total phenolic content of mushroom extracts. It must be noted that this reagent does not react exclusively with phenolics, but other reducing agents, for example, ascorbic acid as well [19, 20]. Hence, results of this test therefore reflect the total reducing capacity of the mushroom extracts and positive controls tested. Despite its tendency to overestimate the level of phenolics, this method is still widely employed prior to quantitative measurement of phenolics using high-performance liquid chromatography (HPLC) [21]. As shown in Table 1, total phenolic content of mushroom extracts tested varied from 6.19 to 63.51 mg GAE/g extract with *G. lucidum* having the highest phenolic content (63.51 ± 1.11 mg GAE/g extract).

Phenolic acids were reported to be the main phenolic compounds in mushrooms [21]. According to Puttaraju et al. [17], gallic acid, tannic acid, protocatechuic acid, and gentisic acids were some of the major phenolics detected in water extracts of several indigenous edible mushrooms from India. Besides, several authors have reported the correlation between the polarity of extraction solvent and phenolic content of resulting extracts [17, 22]. For instance, the phenolic content of *H. erinaceus* hot water extract in this study was determined to be 10.20 ± 2.25 mg GAE/g extract, which was higher than that of methanolic extracts of fresh (0.26 mg GAE/g extract), oven-dried (2.37 mg GAE/g extract), and freeze-dried fruiting bodies (0.78 mg GAE/g extract) of the same species [23].

### 3.2. Antioxidant Capacity of Mushrooms

There is no single, universal method capable of providing an accurate, comprehensive picture of antioxidant profile because several mechanisms underlying antioxidant activity have been proposed including termination of free radical mediated chain reaction, hydrogen donation, chelation of catalytic ions, and elimination of peroxides [24]. Thus, a single assay is not sufficient to measure the total antioxidant capacity of the mushrooms. Moon and Shibamoto [25] recommended that a combination of electron scavenging and lipid peroxidation assays be used. The use of simplified model systems that closely resemble the main features of a given food system was suggested by Kulišić et al. [26]. Multiple assays based on different antioxidant mechanisms are crucial in providing a more reliable approach to assess the antioxidant capacity of the mushrooms. In the present study, a total of five methods—DPPH free radical scavenging activity, *β*-carotene bleaching, inhibition of lipid peroxidation, reducing power ability, and CUPRAC— were used.

### 3.3. Scavenging Effect on DPPH Free Radicals

One of the most common methods for determination of antioxidant capacity is the DPPH free radical scavenging activity assay which relies on the reduction of methanolic DPPH solution in the presence of a hydrogen donating compound (antioxidant). The resulting decolourisation upon absorption of hydrogen from the antioxidant is stoichiometric with respect to the degree of reduction and the remaining DPPH, measured after a certain time, corresponds inversely to the radical scavenging activity of the antioxidant [27]. 

As shown in Table 2, the scavenging activity of mushroom extracts towards DPPH free radicals was expressed in terms of IC_50_. Since a lower IC_50_ value indicates stronger ability of the extracts to act as DPPH radical scavengers, then it is obvious that the positive controls were excellent DPPH radical scavengers with quercetin exhibited >100-fold higher scavenging activity (IC_50_ = 0.03 mg/mL) compared to the mushroom extracts (IC_50_ = 5.28–39.05 mg/mL). Investigated mushrooms showed DPPH free radical scavenging activity, acting possibly as primary antioxidants as suggested by Tsai et al. [11]. *G. lucidum *exhibited significant radical scavenging activity with IC_50_ of  5.28 mg/mL, followed by *Agrocybe *sp. and *P. eryngii* having IC_50_ of 9.56 and 15.42 mg/mL respectively. Apparently, *F. velutipes *displayed the weakest scavenging activity with an IC_50_ of 39.05 mg/mL. 

The ability of hot water extract of other mushrooms to quench free radicals has been reported earlier. Hot water extracts of mature and baby Ling chih (*Ganoderma tsugae* Murrill) showed excellent antioxidant activities with low IC_50_ of 0.30 and 0.40 mg/mL, respectively [10]. In their study, Chirinang and Intarapichet [28] noticed that the radical scavenging activity of water extract of *Pleurotus ostreatus* (IC_50_ = 11.56 mg/mL) was better than that of *P. sajor-caju* (IC_50_ = 13.38 mg/mL) probably due to higher content of phenolic compounds and dietary fibres. *Agaricus blazei*, *Agrocybe cylindracea,* and *Boletus edulis* displayed moderate DPPH scavenging activities with IC_50_ of 13.75, 26.98, and 15.78 mg/mL, respectively [11]. It has been reported that the IC_50_ of hot water extract of *Hypsizygus marmoreus* was 4.19 mg/mL [29] whereas the white mutant strain of the same species was less effective with an IC_50_ of 18.85 mg/mL [30].

### 3.4. *β*-Carotene Bleaching Activity

For evaluation of antioxidant capacity of compounds in emulsions, the *β*-carotene bleaching assay is recommended. Food systems usually comprise of multiple phases where lipid and water coexist with some emulsifiers [31] so it may be feasible to include this assay in our study. Briefly, oxidation of linoleic acid in the emulsion generated radicals which in turn caused the loss of the yellow colour of *β*-carotene. The presence of antioxidants will minimize the extent of *β*-carotene bleaching by neutralizing the radicals [32].

The antioxidant capacities of the mushroom extracts as determined by the *β*-carotene bleaching method were also as expressed as IC_50_ values as shown in Table 2. Low IC_50_ values were noted for quercetin and BHA but a relatively higher IC_50_ value for ascorbic acid suggests it is a weak antioxidant based on the results of this assay despite the fact it is a well-known, polar antioxidant. Our results are in agreement to the previous reports which pointed out that ascorbic acid did not show its antioxidant activity under similar assay [31]. Such phenomenon was termed as “polar paradox” whereby polar antioxidants remaining in the aqueous phase of the emulsion are more diluted in lipid phase and thus, they are less effective [32].

Comparison of *β*-carotene bleaching activity of mushrooms with the positive controls revealed that *S. commune* (IC_50_=2.21 mg/mL) showed comparable activity with quercetin (IC_50_ = 1.86 mg/mL) and even more potent than BHA (IC_50_ = 2.76 mg/mL). Apart from that, three mushroom species, namely, *G. lucidum*, *H. erinaceus,* and *L. edodes* (IC_50_=7.94–8.76 mg/mL) were more effective than ascorbic acid (IC_50_=11.50 mg/mL). The antioxidant activity of *F. velutipes* was the weakest, as evidenced by an IC_50_ that is approximately 20-fold higher than that of *S. commune* and quercetin. 

Apparently, some wild mushrooms demonstrated better inhibition activity in similar assay. Several Portuguese wild mushrooms were reported to show good antioxidant activities as evidenced by IC_50_ in the range of 0.71 to 7.48 mg/mL except for *Hydnum repandum *which had a higher IC_50_ of 28.72 mg/mL [33]. In the concentration of 4–20 mg/mL, water extract of *Lentinula edodes* (52.7–75.9%) showed better antioxidant activities than that of *V. volvaceae* (31.8–65.2%) [22].

### 3.5. Inhibition of Lipid Peroxidation of Buffered Egg Yolk

Lipid peroxidation induced by free radical species results in break down of membrane integrity, affecting its fluidity and permeability [1]. The initial step, that is, peroxidation of polyunsaturated fatty acid components in low-density lipoproteins of membrane produces several byproducts which can damage biomolecules. Transition ions may either generate hydroxyl radicals to initiate the lipid peroxidation process or propagate the chain process via decomposition of lipid hyperoxides [25].

In our study, the lipid peroxidation induced by Fe^2+^ was estimated by the presence of TBARS. The ability of the mushroom extracts to inhibit peroxidation of phospholipids in egg yolk is included in Table 2. At the concentration of 10 mg/mL, inhibition of lipid peroxidation by the mushroom extracts was moderate (30.54–57.18%) whereas quercetin, BHA, and ascorbic acid showed higher inhibition percentage (77.92–81.93%).* G. lucidum* exhibited highest inhibition of lipid peroxidation of 57.18%, followed by *P. florida *and* A. auricular-judae *with inhibition percentage of 56.58% and 56.41%, respectively. Both *V. volvacea* and *P. flabellatus* have comparable percentage of lipid peroxidation inhibition of 50.09% and 50.00%, respectively.

The ability of mushroom extracts to inhibit lipid peroxidation on rat brain homogenate has been reported by Cheung and Cheung [34] who indicated that the dichloromethane subfraction of *V. volvacea* (IC_50_ = 0.109 mg/mL) and *L. edodes* (IC_50_ = 0.297 mg/mL) were active in inhibiting lipid peroxidation. *Phellinus linteus* was reported to inhibit FeCl_2_-induced lipid peroxidation in rat brain homogenate with an IC_50_ of 0.485 mg/mL [35].

### 3.6. Reducing Power Ability

The ability of mushroom extracts to donate electrons could be evaluated using the reducing power assay. In the presence of antioxidants, the Fe^3+^-ferricyanide complex is reduced to the ferrous form, Fe^2+^ and the latter can be monitored by measuring the formation of Perl's Prussian blue at 700 nm; higher absorbance indicates greater reducing power [16].

The ability of the mushrooms extracts and controls to reduce Fe^3+^ to Fe^2+^ at different concentrations are shown in Table 3. At the concentration of 0.50 mg/ml, the positive controls that is quercetin, BHA, and ascorbic acid exhibited high-reducing capacity of 2.506, 2.405, and 2.380, respectively, which were distinctively higher than that of any mushroom extracts (0.011–0.060). Mushroom extracts showed variable reducing capacity and overall, the reducing capacity of mushroom extracts increased with increasing concentration. Amongst the mushrooms, *G. lucidum* showed marked increase in reducing power ability of 0.063 to 0.453 in the tested concentration of 0.01 to 1.00 mg/mL. At 1.00 mg/mL, the reducing power of *G. lucidum *was 0.453 and this was in accordance to work by Mau et al. [10] who reported that the reducing power of mature and baby Ling chih (*G. tsugae* Murrill) was 0.48 and 0.44, respectively. *Agrocybe* sp. and *P. florida* demonstrated comparative increase in reducing power ability of 0.054–0.193 and 0.053–0.110, respectively. Our results for *Agrocybe* sp. were actually comparable to Tsai et al. [36] who reported that reducing power of hot water extracts of *Agrocybe cylindracea* was 0.22 at 1.00 mg/mL. 

The oyster mushrooms (*Pleurotus* spp.) exhibited relatively low reducing power of 0.040–0.081 and 0.043–0.165 at the concentration of 0.50 and 1.00 mg/mL, respectively. Lee et al. [37] in their study reported that *Pleurotus citrinopileatus* showed better reducing power ability than all *Pleurotus* spp. tested in this study. Similarly, Kim et al. [38] found that *Pleurotus cornucopiae* has better reducing capacity than *Pleurotus ostreatus* and *Pleurotus salmoneostramineus*. The reducing power ability of mushrooms can be attributed to the presence of reductones which reacts with peroxides and certain precursors to halt peroxides formation as well as hydrogen-donating ability of these extracts leading to termination of radical chain reactions.

### 3.7. CUPRAC Assay

The CUPRAC assay utilised copper(II)-neocuproine (CU(II)-Nc) reagent as the chromogenic oxidizing agent. It is based on the measurement of absorbance at 450 nm by the formation of stable complex between neocuproine and copper (I) [16]. CUPRAC of the mushroom extracts was assessed and compared to that of the positive controls. As shown in Table 4, CUPRAC of the mushroom extracts was dependent on the concentration of extract. As expected, the positive controls showed more pronounced CUPRAC than the mushroom extracts. At the concentration of 0.50 mg/mL, CUPRAC of quercetin, BHA and ascorbic acid were 2.110, 1.958, and 1.791, respectively. At similar concentrations, *G. lucidum* exhibited significantly higher CUPRAC of 1.058 than other mushroom species. To the best of our knowledge, this is the first report on evaluation of antioxidant activity of hot water extracts of mushroom fruiting bodies by the CUPRAC method. Recently, Omar et al. [39] showed the CUPRAC of *Lentinus squarrosulus* mycelial extract (0.50 mg/mL) was 0.20 ± 0.03 which was lower than CUPRAC of hot water extract of some of the mushrooms studied (Table 4).

### 3.8. Antioxidant Index (AI)

Mushroom hot water extracts consist of a mixture of mainly polar compounds; this complicates the detailed investigation of their antioxidant activities. Some mushroom species showed different levels of antioxidant activities in different assays. For example, *Agrocybe* sp. was shown to be a good antioxidant by the DPPH free radical scavenging assay, reducing power and CUPRAC but moderate and even weak antioxidant by the *β*-carotene bleaching assay and inhibition of lipid peroxidation, respectively. Owing to such chemical complexity and difficulty in comparing results from each assay individually, Antioxidant Index (AI) introduced by Puttaraju et al. [17] was constructed to combine the average results of all five assays. This would be a more accurate approach to display the overall antioxidant potential of the mushrooms in terms of average relative percentages, and comparison can be made with quercetin which consistently showed excellent antioxidant activities in all assays. AI aid in ranking the culinary-medicinal mushrooms according to their antioxidant capacities and this information, if coupled with results from other biological activities of interest (e.g., inhibition of ACE), can be useful for health promotion purposes.

As shown in Table 5, *G. lucidum* exhibited highest relative antioxidant potential (30.1%) followed closely by *S. commune* (27.6%). Several mushrooms showed high to moderate antioxidant potential such as *H. erinaceus* (17.7%), *V. volvace*ae (17.4%), *A. auricular-judae* (16.9%), and *T. heimii* (16.4%). Other mushroom species displayed relatively low antioxidant potential. Consistent with poor antioxidant activities noted in most of the assays, *F. velutipes* was ranked lowest (12.0%). AI of oyster mushrooms studied was comparable and decreased in the following order: *P. flabellatus* (18.4%) > * P. florida* (16.6%) >  *P. eryngii* (15.6%) >  *P. cystidiosus,* and *P. sajor-caju* (14.6%). On the other hand, Puttaraju et al. [17] noted the existence of varietal differences in antioxidant activities among *Termitomyces* spp. in which *T. heimii* and *T. mummiform* showed good antioxidant activity whereas poor activity was observed in *T. microcarpus*. Such observation might be attributed to the chemical composition of active compounds in each species.

### 3.9. ACE Inhibitory Activity

The *in vitro* ACE inhibitory activity of mushroom extracts reflects potential *in vivo* antihypertensive effect as previous studies have demonstrated that inhibition of ACE resulted in decreased blood pressure in animal models [40–43]. In the ACE inhibitory assay, HHL acts as the substrate for ACE which catalyses its conversion to hippuric acid and the dipeptide, histidyl-leucine. ACE activity is assumed to be directly related to the extent of hippuric acid release and hence, lower amount of hippuric acid indicates higher inhibitory activity of extracts towards ACE.

The ACE inhibitory activity of culinary-medicinal mushrooms studied was expressed in terms of IC_50_ as shown in Table 6. Our work demonstrated that hot water extract of *G. lucidum* exhibited the best ACE inhibitory activity with IC_50_ of 0.050 mg/mL. This is followed by the oyster mushrooms, *Pleurotus *spp., which showed comparatively lower IC_50_ values compared to other species with their IC_50_ values (in mg/mL) descend in the following order: *P. eryngii* (0.067) >* P. flabellatus* (0.058) >* P. sajor-caju* (0.056) >* P. cystidiosus* (0.054) >* P. florida* (0.050). The close proximity of their IC_50_ values could be attributed to structural or chemical similarity of the ACE inhibitors produced by the species from the same genus. Accordingly, the ACE inhibitory activities of hot water extract of *G. lucidum* and *Pleurotus* spp. were better than water extract of *Tricholoma giganteum* (IC_50_ = 0.31 mg/mL) [40], *Grifola frondosa* (IC_50_ = 0.28–0.31 mg/mL) [5], and several strains of *Pholiota adiposa* (IC_50_ = 0.21–0.95 mg/mL) [41]. Other mushroom species investigated in this study were shown to be relatively weak in inhibiting ACE with IC_50_ in the range of 0.320 to 0.890 mg/mL. 

Evaluation of ACE inhibitory activity of these mushrooms was carried out using only hot water extracts as water extract (hot and cold) of mushrooms were reported to exhibit higher activity than methanol or ethanol extracts [5, 40]. Effectiveness of both hot and cold water extracts is still not conclusive based on their findings. Apparently, it varies according to mushroom species and influenced by factors like temperature and extraction time [5]. It appears that ACE inhibitory activity increased when extraction time was prolonged. In cases where hot water extract showed lower ACE inhibition, denaturation, or inactivation of the bioactive compounds due to exposure to high temperature has been suggested as the most likely reason. Working on the wild medicinal mushroom *Taiwanofungus camphorates*, Liu et al. [42] reported that only methanol extract exhibited ACE inhibitory activity; the hot water extract showed no activity at all. Nevertheless, isolation and purification of ACE inhibitors from hot water extracts of several mushrooms have been reported. Hagiwara et al. [43] identified a sugar-alcohol, D-mannitol (IC_50_ = 3.00 mg/mL) as the active compound of *Pleurotus cornucopiae.* L-pipecolic acid (IC_50_ = 23.7 mg/mL) purified from the hot water extract of *Sarcodon aspratus* moderately inhibited ACE activity as reported by Kiyoto et al. [44].

It is interesting to point out that *G. lucidum *which possess the highest phenolic content also exhibited highest ACE inhibitory activity in our study. Various medicinal properties of *G. lucidum *might be attributed to its significant antioxidant activities as suggested by Lakshimi et al. [45]. The antihypertensive effect of *G. lucidum*, especially in the field of traditional Chinese medicine is well known. In fact, the *in vitro* ACE inhibitory effect of *G. lucidum* has been reported earlier. Starting from the 70% methanol extract, Morigiwa et al. [46] successfully isolated and characterized five new triterpenes in addition to other five known triterpenes. All showed ACE inhibitory activity with ganoderic acid F exhibited the highest ACE inhibitory effect. 

In our study, the hot water extract of *G. lucidum* and *P. florida* gave the lowest IC_50_ value amongst mushrooms studied. Based on these observations, it is possible to suggest that the hot water extract of both mushroom species could possibly contain effective ACE inhibitor or present in high amount as evidenced by high ACE inhibitory activity at low concentration of extracts. The active compound in the extracts could be proteins and we are currently working on the isolation, characterization, and purification of these potential ACE inhibitor proteins. Previously, peptides with ACE inhibitory activity were isolated from water extracts of edible mushrooms with the following amino acid sequences: Val-Ile-Glu-Lys-Tyr-Pro from *G. frondosa* [6] and Gly-Glu-Pro from *T. giganteum *[40].

### 3.10. Antioxidant Capacities of Mushroom Extracts in Relation to Total Phenolics and Other Compounds

Total phenolic content of the mushroom extracts can be related to their antioxidant capacities. Our findings showed that there was a good correlation (R^2^ = 0.8181) between total phenolics and DPPH free radical scavenging activity of the mushroom extracts. This was in accordance to the findings by several authors who reported that total phenolic content correlated with the free radical scavenging activity of other mushrooms [17, 20]. Total phenolics also showed strong correlation with antioxidant capacity determined by the reducing power assay (R^2^ = 0.8546) and CUPRAC (*R* ^2^ = 0.8279), suggesting that the reductive potential of the mushroom extracts is mainly due to phenolic compounds. In fact, antioxidant activity was shown to be concomitant with the reducing capacity, indicating that antioxidant capacity of the mushroom extracts might be due to its reducing capacity [11]. High free radical scavenging activity, reducing power ability and CUPRAC of some mushrooms, for example, *G. lucidum* may be attributed to high phenolic content as evidenced by findings from this study. According to Dorman et al. [24], the ability of any extracts to reduce Fe^3+^ (a pro-oxidant metal ion) suggests that they may act as free radical chain terminators and transform reactive free radical species into more stable nonradical products. This is in accordance with Puttaraju et al. [17] who reported the strong correlation (R^2^ ~ 0.95) between the phenolics and reducing power ability of several indigenous mushrooms in India. They further suggested that the variation in total antioxidant activity might be attributed to the combinational effect of different phenolic compounds. Based on the close relationship of total phenolics and antioxidant capacities determined by the three assays mentioned above, it might be feasible to suggest phenolics as the major antioxidant components in the mushroom extracts, contribute directly to antioxidative action via radical scavenging ability.

However, the weak correlation between total phenolics and antioxidant capacities as determined by the inhibition of lipid peroxidation and *β*-carotene bleaching assays could imply that phenolics do not act as antioxidants under these conditions. The possibility of active constituents, which are nonphenolic in nature, present in some mushroom extracts must be taken into consideration, for example, total phenolics in *S. commune* was low but the extracts showed great inhibitory action towards *β*-carotene bleaching (IC_50_ = 2.21 mg/mL). Similarly, *A. auricular-judae* and *P. florida* was among the mushrooms with remarkable inhibitory effect on lipid peroxidation, yet total phenolics in both mushrooms were very low. Perhaps, the antioxidant capacities observed could be attributed to other compounds with reducing capabilities, for example, polysaccharides which can be extracted into the hot water extracts. Antioxidative effect of polysaccharides from mushrooms has been reported recently [47].

Chemical constituents in hot water extracts of several mushrooms have been reported earlier, providing insights into the spectrum of potential antioxidant compounds [10, 11, 29, 36, 37]. Ascorbic acid, tocopherols, and *β*-carotene have been reported to be some of the naturally occurring antioxidant compounds in mushrooms [21]. It has been suggested that these compounds may not be extracted into the hot water extracts due to the heat-labile characteristic of ascorbic acid as well as fat-soluble nature of tocopherols and *β*-carotene [37]. Nevertheless, there were reports on detection of ascorbic acid and tocopherols in very low amount from hot water extracts of several mushrooms [10, 11, 29, 36, 48]. Identification of bioactive compounds conferring antioxidant activities would be useful as pure compounds might exhibit higher activity. However, the possibility of various compounds working in a synergistic manner cannot be excluded and in this case, the entire water-soluble fractions (decoctions) are more precious than isolated constituents [49].

Our results also indicated that there was no correlation between ACE inhibitory activity and total phenolics. Based on the literature, ACE inhibitors isolated from mushrooms are mainly peptides [5, 40]. In these cases, these bioactive peptides might block the active site of ACE via substrate-enzyme binding, inhibiting the conversion of angiotensin I to angiotensin II, a potent vasopressor. Apart from inhibition of ACE, blood-pressure-lowering property of these mushroom extracts might be due to other mechanisms such as modulation of nitric oxide production, enhancement of endothelial function, and scavenging of free radicals [7]. Further work is needed to identify potential bioactive compounds responsible for the observed antioxidant and ACE inhibitory activities.

## 4. Conclusions

Culinary-medicinal mushrooms studied showed good antioxidant capacity and potential antihypertensive effect as demonstrated by their inhibitory effect towards ACE. Antioxidants work better in teams, having a broader spectrum offers more complete protection considering the fact that each antioxidant compound works differently and joint effort creates synergy. Therefore, mushrooms in human diets might serve as possible protective agents to help us reduce oxidative damage and the risk of cardiovascular diseases. However, *in vitro* findings are of uncertain relevance to the *in vivo *situation in healthy humans. More work is required to investigate the effects by monitoring a battery of markers *in vivo* oxidative damage or oxidative stress.

## Figures and Tables

**Table 1:** Total phenolic content of culinary-medicinal mushrooms studied.

Mushroom species		Total phenolic content
Scientific name	Common name(s)	(mg GAE/g extract)
*Agrocybe sp.*	Black poplar mushroom; *yangimatusutake* (Japanese)	25.40 ± 1.52^g^
*Auricularia auricular-judae*	Jelly mushroom; Judas's ear fungus; *kikurage* “tree-jelly fish” (Japanese); *mù ěr* “wood ear” (Chinese)	6.19 ± 0.87^a^
*Flammulina velutipes*	Golden needle mushroom; *enokitake* (Japanese); $j\overset{̅}{\iota }nzh\overset{̅}{e}ng$ *ū * (Chinese)	16.69 ± 2.62^d,e^
*Ganoderma lucidum*	*Reishi* (Japanese); *l* $\overset{̅}{\iota }$ *ngzhī* (Chinese)	63.51 ± 3.11^h^
*Hericium erinaceus*	Lion's mane mushroom; *yamabushitake* “mountain-hidden mushroom” (Japanese); *hóutóugū* “monkey head mushroom” (Chinese)	10.20 ± 2.25^a,b^
*Lentinula edodes*	Forest mushroom, *shiitake* “shii mushrooms” (Japanese); *xiānggū* “fragrant mushroom” (Chinese)	14.70 ± 3.01^c,d^
*Pleurotus cystidiosus*	Abalone oyster; summer oyster mushroom	9.26 ± 0.77^a,b^
*Pleurotus eryngii*	King oyster; king trumpet mushroom; French horn mushroom; *eringi* (Japanese); *xìngbàogū* “almond abalone mushroom” (Chinese)	20.95 ± 2.39^f^
*Pleurotus flabellatus*	Pink oyster mushroom	20.24 ± 0.68^e,f^
*Pleurotus florida*	White oyster mushroom	12.24 ± 1.17^b,c^
*Pleurotus sajor-caju*	Grey oyster mushroom	17.70 ± 2.12^d,e,f^
*Schizophyllum commune*	Bracket fungus; spilt-gill fungus; *liezhejun* (Chinese)	16.47 ± 0.42^d,e^
*Termitomyces heimii*	“Termite nest fungus”	11.31 ± 0.42^b,c^
*Volvariella volvaceae*	Paddy straw mushroom; *fukurotake* (Japanese); *cǎogū* (Chinese)	20.88 ± 3.13^f^

Positive controls		

Quercetin		194.24 ± 7.58^i^
Butylated hydroxyanisole (BHA)		931.86 ± 49.78^k^
Ascorbic acid*		25.40 ± 1.39^g^

Values were expressed as mean ± standard deviation of three replicate determinations.

Mean values in a column with different lowercase letters (a–k) indicate significant difference at *P* < .05.

*The value is an estimation of total reducing capacity of ascorbic acid instead of its phenolic content.

**Table 2:** Antioxidant capacities of selected culinary-medicinal mushrooms as determined by the DPPH free radical scavenging activity, inhibition of lipid peroxidation using buffered egg yolk and *β*-carotene bleaching assays.

	DPPH free radical scavenging activity	*β*-carotene bleaching assay	Lipid peroxidation
Mushroom species	IC_50_ (mg/mL)	IC_50_ (mg/mL)	Percentage of inhibition at 10 mg/mL
*Agrocybe *sp.	9.559 ± 0.462^a,b,c^	12.95 ± 0.842^e^	33.33 ± 18.14^a^
*Auricularia auricular-judae*	23.916 ± 0.106^d,e,f^	27.82 ± 0.498^g^	56.41 ± 2.74^c,d^
*Flammulina velutipes*	39.050 ± 5.717^h^	38.80 ± 0.039^h^	41.71 ± 4.23^a,b,c^
*Ganoderma lucidum*	5.280 ± 0.263^a,b^	7.94 ± 0.783^b,c^	57.18 ± 14.05^d^
*Hericium erinaceus*	25.471 ± 0.039^c,d,e^	8.76 ± 1.567^b^	47.52 ± 4.87^a,b,c,d^
*Lentinula edodes*	19.093 ± 0.296^c,d,e^	8.33 ± 0.020^b,c,d^	38.29 ± 4.16^a,b^
*Pleurotus cystidiosus*	31.500 ± 0.053^f,g,h^	26.08 ± 2.383^g^	49.83 ± 4.3^b,c,d^
*Pleurotus eryngii*	15.422 ± 0.037^b,c,d^	24.71 ± 0.542^g^	47.86 ± 9.22^a,b,c,d^
*Pleurotus flabellatus*	17.857 ± 0.000^c,d,e^	11.97 ± 0.317^d,e^	50.09 ± 6.07^b,c,d^
*Pleurotus florida*	21.233 ± 0.045^d,e,f^	25.08 ± 0.333^g^	56.58 ± 7.43^c,d^
*Pleurotus sajor-caju*	23.100 ± 0.156^d,e,f^	17.50 ± 0.012^f^	42.99 ± 3.34^a,b,c,d^
*Schizophyllum commune*	35.659 ± 0.055^g,h^	2.21 ± 0.237^a^	36.24 ± 9.41^a,b^
*Termitomyces heimii*	26.839 ± 0.189^e,f,g^	12.79 ± 0.381^e^	46.32 ± 2.73^a,b,c,d^
*Volvariella volvaceae*	17.832 ± 0.020^c,d,e^	17.92 ± 0.197^f^	50.00 ± 1.47^b,c,d^

Positive controls			

Quercetin	0.032 ± 0.007^a^	1.86 ± 0.014^a^	87.35 ± 6.11^e^
Butylated hydroxyanisole (BHA)	0.097 ± 0.012^c^	2.76 ± 0.014^a^	75.13 ± 2.02^e^
Ascorbic acid	0.078 ± 0.005^b^	11.50 ± 0.135^c,d,e^	81.54 ± 1.09^e^

Values were expressed as mean ± standard deviation of three replicate determinations.

Mean values in a column with different lowercase letters (a–h) indicate significant difference at *P* < .05.

**Table 3:** Reducing power of selected culinary-medicinal mushrooms.

Mushroom species	Absorbance values (at 700 nm) of mushroom extracts of different concentrations (mg/mL)			
	0.05	0.10	0.50	1.00
*Agrocybe *sp.	0.060 ± 0.013^c^	0.054 ± 0.014^d,e^	0.084 ± 0.007^a,b^	0.193 ± 0.002^e^
*Auricularia auricular-judae*	0.035 ± 0.010^a,b,c^	0.032 ± 0.003^b,c,d,e^	0.061 ± 0.005^a,b^	0.110 ± 0.008^b,c^
*Flammulina velutipes*	0.011 ± 0.007^a^	0.011 ± 0.007^a^	0.017 ± 0.006^a^	0.056 ± 0.022^a^
*Ganoderma lucidum*	0.029 ± 0.009^a,b,c^	0.063 ± 0.000^e^	0.270 ± 0.007^c^	0.453 ± 0.046^f^
*Hericium erinaceus*	0.017 ± 0.005^a,b,c^	0.030 ± 0.022^b,c,d^	0.023 ± 0.009^a^	0.077 ± 0.019^a,b^
*Lentinula edodes*	0.024 ± 0.050^a,b,c^	0.017 ± 0.011^a,b,c^	0.036 ± 0.003^a,b^	0.073 ± 0.014^a,b^
*Pleurotus cystidiosus*	0.021 ± 0.006^a,b,c^	0.029 ± 0.010^b,c,d^	0.040 ± 0.009^a,b^	0.043 ± 0.009^a^
*Pleurotus eryngii*	0.003 ± 0.013^a,b^	0.012 ± 0.027^a,b^	0.058 ± 0.003^a,b^	0.165 ± 0.041^d,e^
*Pleurotus flabellatus*	0.031 ± 0.013^a,b,c^	0.044 ± 0.013^c,d,e^	0.080 ± 0.018^a,b^	0.151 ± 0.026^c,d,e^
*Pleurotus florida*	0.035 ± 0.040^a,b,c^	0.053 ± 0.009^d,e^	0.070 ± 0.022^a,b^	0.110 ± 0.007^b,c^
*Pleurotus sajor-caju*	0.037 ± 0.044^b,c^	0.034 ± 0.027^b,c,d,e^	0.081 ± 0.026^a,b^	0.144 ± 0.030^c,d^
*Schizophyllum commune*	0.018 ± 0.002^c^	0.021 ± 0.003^d,e^	0.023 ± 0.004^a,b^	0.490 ± 0.017^g^
*Termitomyces heimii*	0.029 ± 0.010^a,b,c^	0.047 ± 0.024^c,d,e^	0.138 ± 0.038^b^	0.193 ± 0.019^d,e^
*Volvariella volvaceae*	0.043 ± 0.008^b,c^	0.045 ± 0.016^c,d,e^	0.117 ± 0.006^a,b^	0.170 ± 0.003^d,e^

Positive controls*				

Quercetin		2.506 ± 0.054^e^		
Butylated hydroxyanisole (BHA)		2.405 ± 0.033^d,e^		
Ascorbic acid		2.380 ± 0.197^d^		

Values were expressed as mean ± standard deviation of three replicate determinations.

Mean values in a column with different lower case letters (a–g) indicate significant difference at *P* < .05.

*Absorbance values for positive controls were measured at the concentration of 0.50 mg/mL.

**Table 4:** CUPRAC of selected culinary-medicinal mushrooms.

Mushroom species	Absorbance values (at 450 nm) of mushroom extracts of different concentrations (mg/mL)				
	0.10	0.50	1.00	5.00	10.00
*Agrocybe *sp.	0.105 ± 0.022^a,b^	0.396 ± 0.039^a^	0.469 ± 0.095^b,c^	2.318 ± 0.047^e^	2.778 ± 0.015^g^
*Auricularia auricular-judae*	0.242 ± 0.040^d^	0.227 ± 0.022^a^	1.105 ± 0.044^e^	1.401 ± 0.312^b,c^	1.739 ± 0.222^a^
*Flammulina velutipes*	0.063 ± 0.024^a^	0.137 ± 0.015^a^	0.318 ± 0.019^a,b^	1.173 ± 0.034^a,b^	1.958 ± 0.037^b^
*Ganoderma lucidum*	0.519 ± 0.020^e^	1.058 ± 0.103^b^	1.740 ± 0.181^f^	2.802 ± 0.046^f^	2.959 ± 0.000^h^
*Hericium erinaceus*	0.173 ± 0.050^b,c,d^	0.253 ± 0.050^a^	0.548 ± 0.056^c^	1.422 ± 0.065^b,c^	2.442 ± 0.120^d,e^
*Lentinula edodes*	0.155 ± 0.046^b,c^	0.249 ± 0.038^a^	0.561 ± 0.023^c^	1.686 ± 0.044^c,d^	2.578 ± 0.018^e,f^
*Pleurotus cystidiosus*	0.185 ± 0.083^c,d^	0.150 ± 0.029^a^	0.487 ± 0.045^b,c^	1.435 ± 0.187^b,c^	2.144 ± 0.132^c^
*Pleurotus eryngii*	0.125 ± 0.017^a,b,c^	0.275 ± 0.013^a^	0.458 ± 0.056^b,c^	1.967 ± 0.034^d,e^	2.657 ± 0.074^f,g^
*Pleurotus flabellatus*	0.146 ± 0.015^b,c^	0.328 ± 0.021^a^	0.530 ± 0.060^c^	1.574 ± 0.082^c^	2.467 ± 0.071^d,e^
*Pleurotus florida*	0.070 ± 0.027^a^	0.165 ± 0.012^a^	0.390 ± 0.072^a,b,c^	1.395 ± 0.129^b,c^	2.345 ± 0.056^d^
*Pleurotus sajor-caju*	0.183 ± 0.026^c,d^	0.208 ± 0.031^a^	0.444 ± 0.041^b,c^	1.520 ± 0.029^b,c^	2.427 ± 0.018^d,e^
*Schizophyllum commune*	0.114 ± 0.009^a,b,c^	0.244 ± 0.046^a^	0.832 ± 0.214^d^	1.476 ± 0.061^b,c^	2.060 ± 0.031^b,c^
*Termitomyces heimii*	0.127 ± 0.014^a,b,c^	0.184 ± 0.013^a^	0.250 ± 0.048^a^	0.819 ± 0.456^a^	2.378 ± 0.066^d^
*Volvariella volvaceae*	0.165 ± 0.031^b,c^	0.303 ± 0.041^a^	0.831 ± 0.078^d^	2.028 ± 0.212^d,e^	2.788 ± 0.015^g,h^

Positive controls*					

Quercetin			2.110 ± 0.227^d^		
Butylated hydroxyanisole (BHA)			1.958 ± 0.470^c,d^		
Ascorbic acid			1.791 ± 0.220^c^		

Values were expressed as mean ± standard deviation of three replicate determinations.

Mean values in a column with different lowercase lower case letters (a–h) indicate significant difference at *P* < .05.

*Absorbance values for positive controls were measured at the concentration of 0.50 mg/mL.

**Table 5:** Grading of selected culinary-medicinal mushrooms for total antioxidant activity according to the method by Puttaraju et al. [17].

	Relative percentage of each antioxidant capacity assay					
Mushroom species/positive controls	DPPH	*β*-CB	ILP	RPA	CUPRAC	Average of total antioxidant activity (Relative percentage = antioxidant index)
(1) Quercetin	100.0	100.0	100.0	100.0	100.0	100.0
(2) BHA	30.5	67.4	86.0	96.0	92.8	74.5
(3) Ascorbic acid	41.6	16.2	93.0	95.0	84.9	66.2
(4)* Ganoderma lucidum *	0.6	23.4	65.5	10.8	50.1	30.1
(5)* Schizophyllum commune *	0.1	84.1	41.5	0.9	11.6	27.6
(6)* Pleurotus flabellatus *	0.2	15.5	57.3	3.2	15.5	18.4
(7)* Hericium erinaceus *	0.1	21.2	54.4	0.9	12.0	17.7
(8)* Volvariella volvaceae *	0.2	10.4	57.2	4.7	14.4	17.4
(9)* Auricularia auricular-judae *	0.1	6.7	64.6	2.4	10.8	16.9
(10)* Pleurotus florida *	0.2	7.4	64.8	2.8	7.8	16.6
(11)* Termitomyces heimii *	0.1	14.6	53.0	5.5	8.7	16.4
(12)* Lentinula edodes *	0.2	22.4	43.8	1.4	11.8	15.9
(13)* Pleurotus eryngii *	0.2	7.5	54.8	2.3	13.0	15.6
(14)* Agrocybe *sp.	0.3	14.4	38.2	3.4	18.8	15.0
(15)* Pleurotus sajor-caju *	0.1	10.6	49.2	3.2	9.9	14.6
(16) *Pleurotus cystidiosus *	0.1	7.1	57.0	1.6	7.1	14.6
(17) *Flammulina velutipes *	0.1	4.8	47.7	0.7	6.5	12.0

The average of DPPH (free radical scavenging activity), *β*-CB (*β*-carotene bleaching assay), ILP (inhibition of lipid peroxidation), RPA (reducing power ability), and CUPRAC (cupric ion reducing antioxidant capacity) of quercetin is based on 100. Antioxidant potential (average of total antioxidant activity) of other positive controls and mushrooms studied was expressed as average relative percentages compared to quercetin.

**Table 6:** ACE inhibitory activity of selected culinary-medicinal mushrooms.

Mushroom species	ACE inhibitory activity
	IC_50_ (mg/mL)
*Agrocybe *sp.	0.890 ± 0.046
*Auricularia auricular-judae*	0.510 ± 0.018
*Ganoderma lucidum*	0.050 ± 0.009
*Hericium erinaceus*	0.580 ± 0.023
*Pleurotus cystidiosus*	0.054 ± 0.002
*Pleurotus eryngii*	0.067 ± 0.026
*Pleurotus flabellatus*	0.058 ± 0.002
*Pleurotus florida*	0.050 ± 0.013
*Pleurotus sajor-caju*	0.056 ± 0.012
*Schizophyllum commune*	0.320 ± 0.070
*Volvariella volvaceae*	0.760 ± 0.023

IC_50_ values were interpolated from dose-response curve for ACE inhibition of each mushroom species. Values were expressed as mean ± standard deviation of three replicate determinations.
